# Resection of intrahepatic cholangiocarcinoma in elderly patients – is it reasonable?

**DOI:** 10.1186/s12893-019-0620-7

**Published:** 2019-10-29

**Authors:** Fabian Bartsch, Janine Baumgart, Verena Tripke, Maria Hoppe-Lotichius, Stefan Heinrich, Hauke Lang

**Affiliations:** Department of General, Visceral and Transplant Surgery, University Medical Center of the Johannes Gutenberg-University Mainz, Langenbeckstraße 1, 55131 Mainz, Germany

**Keywords:** Intrahepatic cholangiocarcinoma, Cholangiocarcinoma, Liver surgery, Geriatric, Elderly

## Abstract

**Background:**

Intrahepatic cholangiocarinoma (ICC) has a rising incidence in western countries. Often major or extended resections are necessary for complete tumor removal. Due to demographical trends the number of elderly patients diagnosed with ICC is rising accordingly. Aim of this study is to show whether resection of ICC in elderly patients is reasonable or not.

**Methods:**

Between January 2008 and June 2018 all consecutive patients with ICC were collected. Analyses were focussed on the performed resection, its extent, postoperative morbidity and mortality as well as survival. Statistics were performed with Chi^2^ test for categorical data and for survival analyses the Kaplan Meier model with log rank test was used.

**Results:**

In total 210 patients underwent surgical exploration with 150 resections (71.4%). Patients were divided in 70-years cut-off groups (> 70 vs < 70 years of age) as well as a young (age 30–50, *n* = 23), middle-age (50–70, *n* = 76) and old (> 70, *n* = 51) group, whose results are presented here. Resectability (*p* = 0.709), extent of surgery (*p* = 0.765), morbidity (*p* = 0.420) and mortality (*p* = 0.965) was comparable between the different age groups. Neither visceral (*p* = 0.991) nor vascular (*p* = 0.614) extension differed significantly, likewise tumor recurrence (*p* = 0.300) or the localisation of recurrence (*p* = 0.722). In comparison of patients > or < 70 years of age, recurrence-free survival (RFS) was significantly better for the younger group (*p* = 0.047). For overall survival (OS) a benefit could be shown, but without reaching significance (*p* = 0.072). In subgroup analysis the middle-age group had significant better OS (*p* = 0.020) and RFS (*p* = 0.038) compared to the old group. Additionally, a better OS (*p* = 0.076) and RFS (*p* = 0.179) was shown in comparison with the young group as well, but without reaching significance. The young compared to the old group had analogous OS (*p* = 0.931) and RFS (*p* = 0.845).

**Conclusion:**

Resection of ICC in elderly patients is not associated with an increased perioperative risk. Even extended resections can be performed in elderly patients without obvious disadvantages. Middle-age patients have a clear benefit for OS and RFS, while young and old patients have a comparable and worse long-term outcome.

## Background

Intrahepatic cholangiocarcinoma (ICC) has a rising incidence over the last decades, especially in western countries [1–3]. Beside younger patients who are noticeable more often diagnosed with ICC from our own experience, also elderly or geriatric patients are referred to tertiary centers more often for evaluation of resection [4]. Social demographic trend in Europe also develops to an older society [5]. Elderly patients who need to undergo liver surgery should be selected wisely, individualized approaches and multidisciplinary postoperative care are important [6]. Frailty assessment through scoring systems or excluding other negative predictors like cirrhosis might help to optimise postoperative outcome [7, 8]. Further an ASA-score of 3 or 4 seems to be predictive for postoperative morbidity in elderly patients, especially when the body mass index was > 26 kg/m^2^ in colorectal liver metastases (CRLM) [9]. Data from Taiwan and Japan shows that liver resection in elderly patients is safe and feasible for elderly patients with hepatocellular carcinoma (HCC) as well [10–12]. Age seems to be no contraindication for liver resection. For ICC data regarding this special topic is scarce. Vitale and colleagues showed in a multi-center study that elderly patients with ICC had an increased risk for perioperative complications, but with comparable overall and recurrence-free survival [13].

Aim of this study is to analyse the feasibility and risks of surgical resection of ICC in elderly patients in a single-center collective with predominant major and extended liver resections. Extent of resection, morbidity, mortality and long-term outcome will be focussed in analysis.

## Methods

All consecutive explorations and resections for patients with ICC were collected in a prospective institutional database in between January 2008 and June 2018. Perihilar and distal cholangiocarcinoma, gallbladder carcinoma, hepatocellular carcinoma and all secondary liver malignancies were excluded. If a tumor was centrally located with contact and/or infiltration of the liver hilum exceeding a diameter of 3 cm and an obvious origin of secondary or tertiary bile ducts (in preoperative imaging and/or histologically), the tumor was included as ICC.

All patients signed informed consent that data and follow-up will be collected anonymously and is potentially used for scientific analysis. Regarding to the regulations of the federal state law (state hospital law §36 & §37) and the independent ethics committee of Rhineland-Palatinate, no ethical approval was necessary for this study.

### Preoperative work-up, surgical procedures and follow-up

For preoperative evaluation and planning a high-resolution computed tomography (CT) or magnetic resonance imaging (MRI) of good quality was inevitable. Most patients were referred from secondary centers with already histologically proven or the suspicion of ICC. If metastatic disease of a gastrointestinal malignancy has not been ruled out through the referring center we performed gastroscopy and colonoscopy.

Experienced hepato-pancreatico-biliary surgeons performed all explorations and/or liver resections. Minor resections were defined as ≤3, major resections as 4 resected segments. Resections with ≥5 liver segments were classified as extended resection likewise all mesohepatectomy, associating liver partition and portal vein ligation for staged hepatectomy (ALPPS) and major or minor resections with visceral and/or vascular extension.

Regular follow-up was performed every 3 months at least for 2 years after primary resection with ultrasound, CT scan or MRI. Thereafter, it was continued every 6 months. Due to distance some patients were not able to undergo follow-up at our center. In these cases, we contacted the referring physician to get all necessary information.

### Data analysis

Special focus was patients’ age, extent of resection, morbidity and mortality as well as tumor recurrence and survival. Morbidity was classified according to the Dindo-Clavien classification [14]. Mortality is provided as 30-day and 90-day mortality. All postoperative in-hospital deaths occurred in these time range. Recurrence-free survival was defined after Punt et al. [15].

### Statistical analysis

For statistics data was transferred into a SPSS 23 database (SPSS Inc. Released 2014, IBM SPSS Statistics for Windows, Version 23.0, IBM Armonk, NY, USA). Only patients with complete data-sets were included in analysis. *P*-values < 0.05 were considered significant. For categorical data Chi^2^-test was utilized. For analysis and comparison of overall survival and recurrence-free survival the Kaplan Meier model with log rank test was used. For multivariate analyses (predictors for survival) the Cox regression (proportional hazards model) was used. Significant parameters out of the univariate analyses were analysed using backward selection and age was included for overall survival even if it did not reach significance.

## Results

We report on a cohort of 210 patients with ICC (102 women and 108 male) with 150 who underwent resection. Median age was 64.2 years (IQR: 56.2–74.1; range 32.3–84.4 years). Sixty tumors were irresectable at exploration due to peritoneal carcinomatosis (*n* = 23), multifocal tumor dissemination (*n* = 15), locally advanced infiltration (*n* = 11) or cirrhosis/small for size liver remnant/poor quality of parenchyma (n = 11).

### Age distribution and surgical procedures

Distribution of patients’ age is listed for detailed subgroups in Table 1 with further information on extent of surgery, morbidity, mortality and survival. We had two different groupings: First a 70-year cut-off with patients younger or older than 70 years, and second different age groups with categories “young” (30–49.9 years), “middle-age” (50–69.9 years) and “old” (70–90 years; see also Table 1).

**Table 1:** Extent of resection, morbidity, mortality and long-term outcome according to patients’ age distribution

	< 70	>70	Young 30–49.9	Middle-age 50–69.9	Old > 70	All	%
Resection^a^	99	51	23	76	51	150	
Extended resection	57	30	16	41	30	87	58
Major resection	17	9	3	14	9	26	17.3
Minor resection	25	12	4	21	12	37	24.7
Exploration	36	24	8	28	24	60	
Resection rate	73.3%	68%	74,2%	73.1%	68%	71.4%	
Morbidity^b^							
no morbidity	51	30	13	38	30	81	54
Grade I or II	10	7	–	10	7	17	11.3
Grade IIIa	19	9	5	14	9	28	18.7
Grade IIIb	2	1	–	2	1	3	2
Grade IV a	6	–	3	3	–	6	4
Grade IVb	2	–	–	2	–	2	1.3
Mortality (Grade V)	9	4	2	7	4	13	8.7
Overall survival ^c^							
Median (months)	27.2	20.2	19.3	30.1	20.2	23.6	
1-year	80%	76%	74%	82%	76%	79%	
3-year	37%	23%	27%	40%	23%	32%	
5-year	20%	11%	13%	22%	11%	17%	
Recurrence-free survival ^c^							
Median (months)	10.5	8.4	9	11	8.4	9.7	
1-year	43%	28%	33%	45%	28%	38%	
3-year	21%	8%	7%	24%	8%	16%	
5-year	15%	8%	–	17%	8%	12%	

^a^extended resections were all resection ≥5 segments, ALPPS, mesohepatectomy and all resections with visceral and/or vascular extensions, major resections were resections of 4 segments (all hemihepatectomies), minor resections were all ≤3 segments; ^b^ highest morbidity of resection group; ^c^ only resection group, perioperative deaths were excluded

Trisectionectomy was most frequent with 48 (right *n* = 26, left *n* = 22) and followed by hemihepatectomy with 44 resections (right *n* = 25, *n* = 19). Mesohepatectomy was performed seven times, associating liver partitioning and portal vein ligation for staged hepatectomy (ALPPS) six times. The age categories had no influence on resectability (*p* = 0.412 for first and *p* = 0.709 for second groupings) or extent of surgery (*p* = 0.973 and *p* = 0.765).

Visceral and/or vascular extensions were performed in 66 patients with 44 visceral and 35 vascular extensions (13 patients underwent both). Neither visceral and/or vascular extension together (*p* = 0.374) nor visceral (*p* = 988) or vascular (*p* = 0.392) extension differed significantly between the 70-years cut-off groups as well as the different age groups (*p* = 0.525, *p* = 0.991 and *p* = 0.614, respectively).

### Morbidity and mortality

Detailed morbidity is listed in Table 1. All perioperative deaths occurred within 90-days after surgery (30-day *n* = 12, 90-day mortality n = 1) and no further in-hospital death has been recorded. Reasons for perioperative deaths were multiorgan-failure (*n* = 6), liver failure (*n* = 4) or sepsis (*n* = 3). The 70-year cut-off as well as the different age groups had no influence on morbidity (*p* = 0.188 and 0.420) or mortality (*p* = 0.797 and *p* = 0.965).

### Tumor recurrence and recurrence-free survival

Tumor recurrence occurred in 97 patients (64,7%) with localisation intrahepatic only in 41 (42.3%), extrahepatic only in 25 (25.8%) and combined intra- and extrahepatic in 31 (31.9%) patients. Occurrence of recurrence did not differ significantly between the 70-years cut-off groups (*p* = 0.201) or the different age groups (*p* = 0.300). Similarly, the localisation of recurrence did not differ (*p* = 0.371 for 70-years cut-off groups and *p* = 0.722 for different age groups).

**Fig. 1:**
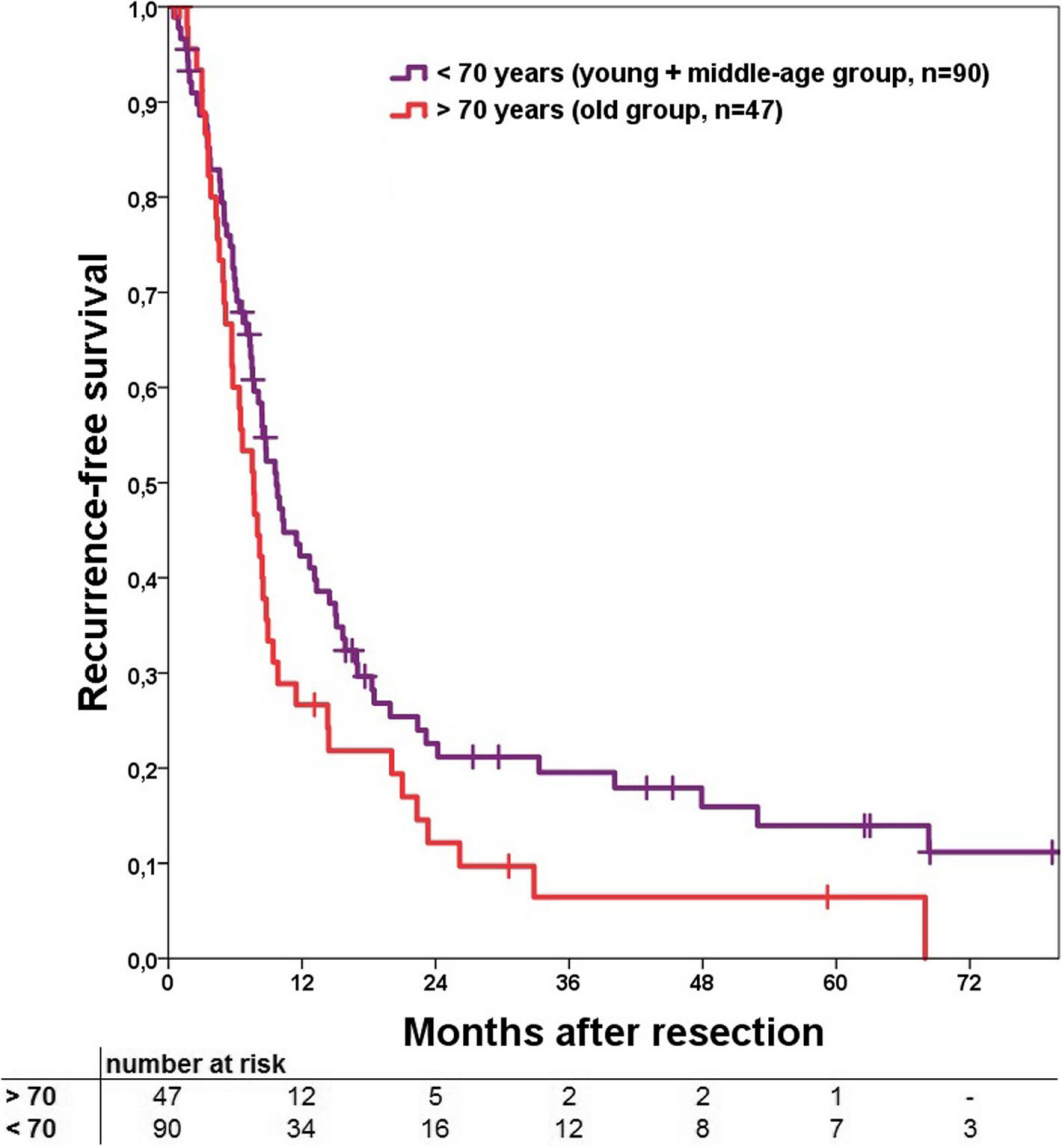
Kaplan Meier curve to compare recurrence-free survival for the > 70 and < 70 years of age groups. No significant difference could be shown (*p* = 0.072). Perioperative deaths were excluded

**Fig. 2:**
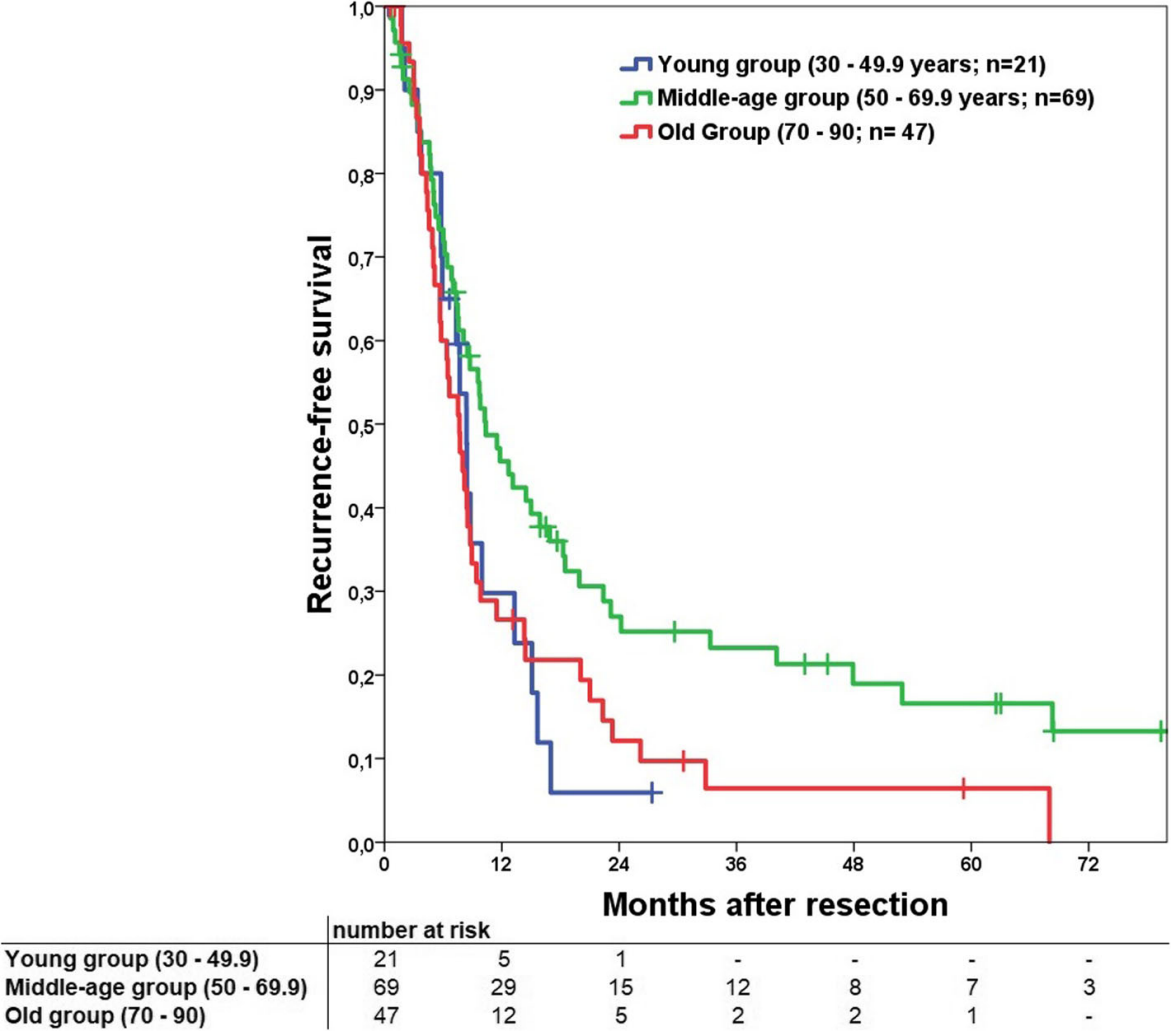
Kaplan Meier curve to compare recurrence-free survival for the young, middle-age and old groups. Overall a significant difference can be shown (*p* = 0.034). Comparison of the isolated subgroups demonstrates a significant difference in comparison of middle-age vs. old group (*p* = 0.020). No differences were found for young vs. middle-age (*p* = 0.076) and young vs. old groups (*p* = 0.931). Perioperative deaths were excluded

Median recurrence-free survival (RFS) was 9.7 months. Consecutive 1-, 3- and 5-year RFS was 38, 16 and 12%, respectively. RFS for the 70-years cut-off groups showed a significant difference (Fig. 1, *p* = 0.047) as well as for the three different age groups (Fig. 2, *p* = 0.034). In subgroup comparison the young and old group had a comparable RFS (*p* = 0.931) while the middle-age group had a significant better RFS compared to the old group (*p* = 0.020).

### Overall survival

**Fig. 3:**
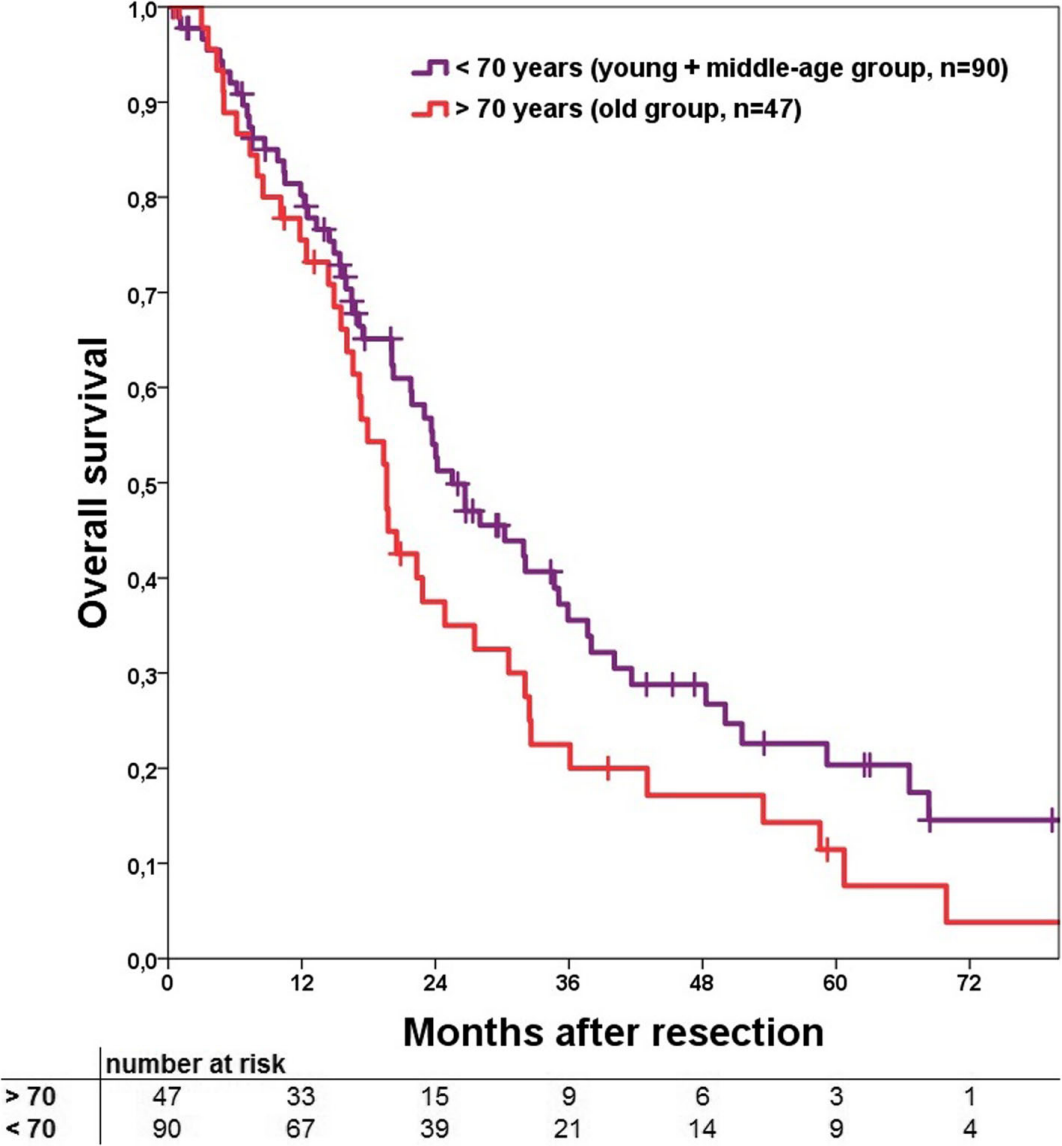
Kaplan Meier curve to compare overall survival for the > 70 and < 70 years of age groups. A significant difference could be shown (*p* = 0.072). Perioperative deaths were excluded

**Fig. 4:**
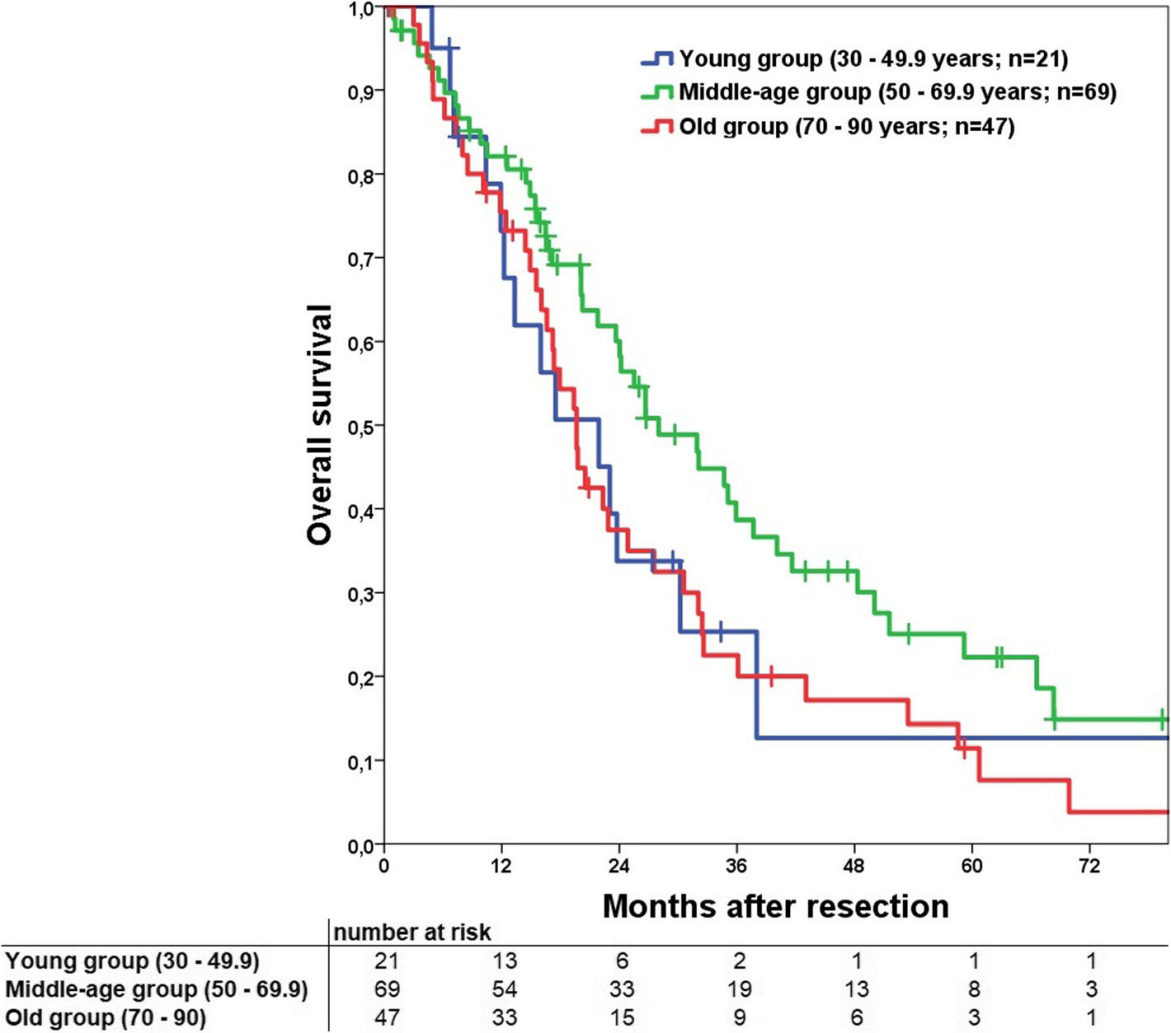
Kaplan Meier curve to compare overall survival for the young, middle-age and old groups. Overall no significant difference can be shown (*p* = 0.094). Comparison of the isolated subgroups demonstrates a significant difference in comparison of middle-age vs. old group (*p* = 0.038). No differences were found for young vs. middle-age (*p* = 0.179) and young vs. old groups (*p* = 0.845). Perioperative deaths were excluded

Median overall survival (OS) for the resection group with perioperative deaths excluded was 23.6 months. Consecutive 1-, 3- and 5-year OS was 79, 32 and 17%, respectively. The comparison of OS on the 70-years cut-off groups showed no significant difference (Fig. 3, *p* = 0.072) as well as the three different age groups in general (Fig. 4, *p* = 0.094). In comparison of the subgroups the middle-age group had a significant better OS compared to the old group (*p* = 0.038) with no significant difference for the two other combinations.

### Factors influencing or predicting survival

Different histopathological factors and their distribution in the 70-years cut-off groups as well as the three different age groups are shown in Table 2. Further statistical analyses showed that only N-stage differed statistically significant in both age groups with N1 status being less frequent in the > 70 years group. Other factors were comparable between the different age groups.

**Table 2:** Histopathological factors and their distribution within different age groups

		<70 *n* = 99	> 70 *n* = 51	*p*-value	Young < 50 *n* = 23	Middle-age 50–70 *n* = 76	Old > 70 *n* = 51	*p*-value
Tumor size^a^ (cm)	Mean Median (IQR)	7.76 7 (4.6–10.5)	7.38 6.75 (5–9)		8.45 7.5 (6.2–11)	7.55 6.5 (4.5–10)	7.38 6.75 (5–9)	
	≤ 10 / > 10 cm	69 / 28	40 / 10	0.245	15 / 8	54 / 20	40 / 10	0.386
	≤ 5 / > 5 cm	26 / 71	12 / 38	0.713	4 / 19	22 / 52	12 / 38	0.466
Multifocality	yes / no	35 / 64	11 / 40	0.083	12 / 11	52 / 24	40 / 11	0.074
Number of nodules^b^	Mean	2.1	1.56		2.86	1.86	1.56	
	≤3 / ≥4	79 / 16	45 / 4	0.154	16 / 6	63 / 10	45 / 4	0.098
T-stage	T1 + T2 / T3 + T4	72 / 27	40 / 11	0.447	14 / 9	58 / 18	40 / 11	0.246
N-stage^c^	N0 / N1	53 / 35	37 / 8	**0.010**	13 / 9	40 / 26	37 / 8	**0.037**
R-stage	R0 / R1	86 / 13	45 / 6	0.812	20 / 3	66 / 10	45 / 6	0.972
Grading	G1 + G2 / G3 + G4	59 / 32	36 / 12	0.221	14 / 8	45 / 24	36 / 12	0.468

^a^size of biggest nodule, missing in 3 cases; ^b^ exact number of nodules missing in 6 cases; ^c^ 19 patients with Nx; significant *p*-values are bold

Histopathological factors were tested for their influence on OS and RFS (Table 3). Significant factors of the univariate analysis were further included in multivariate analyses as well as age with the 70-years cut-off, even if not significant for OS. For OS N-stage and tumor size (with 10 cm cut-off) showed to be independent predictors. For RFS tumor size, N-stage, age (70-years cut-off) and multifocality predicted independently (Table 3).

**Table 3:** Univariate and multivariate analyses

		Kaplan Meier Model		Multivariate Cox regression model					
		OS	RFS	OS			RFS		
				HR	95% CI	*p*-value	HR	95% CI	*p*-value
Age	> 70 / < 70	0.072	**0.047**	1.440	0.929–2.232	0.103	**1.826**	1.179–2.826	**0.007**
Age	> 70 / 50–70 / < 50	0.094	**0.034**			‡^1^			‡^2^
Tumor size	≤5 cm / > 5 cm	0.091	0.009*						
	≤ 10 cm / > 10 cm	**0.013**	**0.002**	1.812	1.100–2.985	**0.020**	2.055	1.294–3.262	**0.002**
Multifocality	yes / no	0.262	**0.014**				1.701	1.101–2.629	**0.017**
T-stage	T1 + T2 / T3 + T4	0.102	0.347						
N-stage	N0 / N1	**0.003**	**0.026**	2.121	1.336–3.367	**0.001**	1.877	1.211–2.908	**0.005**
V-stage	V0 / V1 + V2	0.149	0.818						
L-stage	L0 / L1	0.369	0.673						
Pn-stage	Pn0 / Pn1	**0.027**	0.091	1.432	0.889–2.308	0.140			
R-stage	R0 / R1	0.655	0.254						
Grading	G1 + G2 / G3 + G4	0.248	0.736						

Perioperative deaths were excluded for statistical analyses; significant parameters are bold; 70 years cut-off groups were included in multivariate analyses for OS even if not significant (underlined); ‡ if this age groups were included instead in multivariate analysis, age was eliminated within the first step with a *p*-value of 0.501 for ‡^1^ (OS) and 0.103 for ‡^2^ (RFS); * Only one significant tumor size cut-off was included in multivariate analyses

*OS* overall survival, *RFS* recurrence-free survival, *HR* hazard ratio, 95% *CI* 95% confidence interval

## Discussion

The feasibility of liver surgery in elderly patients is discussed intensively. For patients with ICC in a cohort of mainly extended and major resections, we were able to show that patients’ age had no influence on resectability, extent of surgery or morbidity/mortality. Likewise, tumor recurrence were not more frequent in the elderly group. While the young and old group had a comparable OS and RFS, the middle-age group had a significantly better OS and RFS compared to the old group.

Liver surgery as well as perioperative therapy has evolved. For elderly patients liver resections become increasingly common [16]. Data for resection of colorectal liver metastases (CRLM) shows only a small increase of postoperative morbidity and mortality [17, 18]. Postoperative outcomes differ within the literature. Some authors describe liver surgery in elderly patients as safe as in young [9, 19]. Others see slightly higher morbidity or mortality rates with especially postoperative pneumonia as a risk factor for mortality [20, 21]. Tufo and colleagues were able to show that the number of patients who underwent liver resection older than 70 years raised from 6% in 1990 to > 25% in 2007 [18]. Parenchyma sparing liver resections got more frequent over the years leading to a decrease of major hepatectomies and subsequently also a decrease in morbidity and mortality.

We were able to show that morbidity and mortality was comparable between our 70-year cut-off groups as well as a grouping of young, middle-age and old patients. The distribution of different grades of complications shows to be largely analogous as well, considering the number of patients within the subgroups. In regard of a cohort with many major and extended resections, our data suggests that liver resection is safe and feasible.

Data for resection of ICC in elderly patients is scarce. To the best of our knowledge Vitale and colleagues were the only authors addressing liver resection for ICC in elderly patients [13]. In a multi-center study they used 70 years of age as cut-off with 129 older and 455 younger patients. Elderly patients had a higher incidence of morbidity as well as major morbidity, while mortality did not differ between both groups. After propensity score matching disease-free survival and OS were comparable between both groups. Tumor characteristics showed to be more predictive regarding long-term outcomes. This data is in accordance to our findings. If we analysed a cut-off of 70 years with an elderly versus a younger group, RFS and OS were comparable as well. Morbidity and mortality did not differ between our groups as mentioned before, which might be affected to the smaller number of patients and subgroups.

In addition to the cut-off of 70 years we further differentiated the <70 years group in <50 and 50–70 years. A very interesting finding was that the young patient group (< 50 years) had a comparable OS and RFS compared to the old patient group, while the middle-age group had a clear benefit over both. Most likely this can be explained by a more aggressive tumor biology in younger patients evading cellular cancer control mechanisms even at young age and leading to a more invasive tumor growth pattern. This data for ICC is very difficult to discuss because Vitale and colleagues divided their analysis in patients > or < 70 years of age, but did not do any further differentiation of the younger group. Therefore, we have no group to compare, and thus no statement can be made for a younger group of patients.

In comparison of different histopathological factors and their distribution within the different age groups, only N-stage showed to be significantly different. In univariate and multivariate analyses typical factors showed to influence OS as well as RFS [22–24]. Age was an independent predictor only for RFS using the 70-year cut-off.

Of course, not every elderly patient qualifies to undergo major or extended liver resection. In a prospective mutli-center trial Tanaka and colleagues used a phenotypic frail index to predict age-related events after liver surgery [8]. They were able to show that frailty and resection of ≥2 sectors were independent risk factors. We had no hard criteria for selection which patient qualified for resection, but used the ASA classification (American Society of Anaesthesiologists) and the WHO/ECOG performance status [25, 26]. An ASA IV patients with a WHO/ECOG performance status of ≥3 might be no candidate for liver surgery. With ASA IV and WHO/ECOG 1 or 2 the risk for postoperative morbidity and mortality is increased. Nevertheless, we go for surgery if the patient explicitly wishes resection after a detailed description of the procedure and accompanied risks.

This study has some limitations. Of course, a bigger number of patients would raise the power of statistical testing, especially in subgroup analysis. Therefore, in our case a multicentre study would be desirable to raise the number of included patients. Our cohort has a very high proportion of extended and major resections. On one hand this is helpful, because it underlines that even major and extended resections are feasible in elderly patients. On the other hand, it makes it even more difficult to compare our data with the scarce data of the literature.

## Conclusions

In conclusion we were able to show that resectability, extent of surgery, morbidity and mortality were not influenced by patients’ age. Incidence of tumor recurrence as well as location of recurrence were comparable between the young, middle-age and old groups. Interestingly young and elderly patients had a comparable OS and RFS, whereas the middle-age group had a significantly better OS and RFS compared to the old group. Liver resection for ICC is safe and feasible in elderly patients and offers a chance of long-term survival.

## Data Availability

The datasets used and analyzed during the current study are available from the corresponding author on reasonable request.

## Abbreviations

ALPPS: Advanced liver partition and portal vein ligation for staged hepatectomy
ASA: American Society of Anaesthesiologists
CRLM: Colorectal liver metastasis
CT: Computed tomography
ECOG: Eastern Co-operative of Oncology Group
HCC: Hepatocellular carcinoma
ICC: Intrahepatic cholangiocarcinoma
IQR: Interquartile range
MRI: Magnetic resonance imaging
OS: Overall survival
RFS: Recurrence-free survival
WHO: World Health Organisation
